# Torkildsen shunt as a salvage procedure for an infant with post-hemorrhagic hydrocephalus

**DOI:** 10.1016/j.ijscr.2018.11.058

**Published:** 2018-11-27

**Authors:** Dimitrios Panagopoulos, Marios Themistocleous, Andreas Mitsios, Georgios Sfakianos

**Affiliations:** Thivon & Papadiamantopoulou St, Goudi, 11527, Athens, Attica, Greece

**Keywords:** VP shunt, ventriculo-peritoneal shunt, CSF, cerebrospinal fluid, CT, computed tomography, MRI, magnetic resonance imaging, EVD, external ventricular drainage, T2 GRE, T2 gradient echo sequence, VCS, ventriculocisternostomy, VA shunt, ventriculo-atrial shunt, CNS, central nervous system, Torkildsen operation, Cisterna magna, Occipital horn

## Abstract

**Introduction:**

Torkildsen operation is a ventriculo-cisternal shunt that diverts the cerebrospinal fluid flow from one of the lateral ventricles, via a ventricular catheter, to the cisterna magna of the posterior fossa. It is the first described operation of CSF diversion for the treatment of hydrocephalus [1] (Scarff, 1963).

**Presentation of case:**

We present the case of a premature infant who presented with post hemorrhagic hydrocephalus who underwent several failed CSF diversion surgeries before Torkildsen shunt procedure. The patient overcame the irregularities of CSF circulation that were observed before the operation and his post-operative course was uneventful, in terms of CSF –related complications.

**Discussion:**

We summarize the historical data and the technical aspects of the procedure, and we present a brief literature review of the indications and limitations associated with it.

**Conclusion:**

Torkildsen shunt can be effective in selected patients with hydrocephalus even in the modern era of computed tomography and magnetic resonance imaging. the procedure enables one to avoid a standard ventriculoperitoneal shunt, especially in cases it is not technically feasible.

## Highlights

• Modern techniques for treatment of infantile post-hemorrhagic hydrocephalus failed.
• This operation is an internal shunt that avoids use of ventriculo-peritoneal shunt.
• It is currently generally regarded as an “obsolete surgical procedure”.

## 1 Introduction

We state that he work has been reported in line with the SCARE criteria, as sited in our references [2]

The Torkildsen shunt was first performed in 1937 by Dr. Arne Torkildsen, a Norwegian neurosurgeon, and reported in 1939 [1,3,4]. It is considered to be a type of internal ventriculocisternal shunt that diverts the cerebrospinal fluid (CSF) flow from one of the lateral ventricles, namely the occipital horn, to the cisterna magna of the posterior fossa.

Although it seems that initially had been widely accepted as efficient surgical procedure for treating hydrocephalus, introducement of the VA shunt and later of the VP shunt procedure, was the cause that it was largely abandoned.

In this paper, we report our experience using this historical procedure to a case of infantile hydrocephalus that seemed to be untreatable with modern therapeutic strategies.

## 2 Presentation of case

We refer to a premature infant, 26 weeks of gestational age at birth, who was born via cesarean incision with sciatic projection. Amniocentesis was executed three days before birth in order to investigate the possibility of short extremities stature, after which mother suffered placental abruption. The infant’s gestational weight was 760 g and he was hospitalized in an incubator.

Two days after birth, as a screening test, an initial ultrasound via the anterior fontanelle was performed, which verified the presence of intraventricular hemorrhage grade IV with accompanying porencephalic cysts that were communicating with the ventricular system. Clinical and neurological examination recognized only an upcoming fullness of the anterior fontanelle and a left posterior plagiocephaly.

From the patient medical history, we have to mention that he suffered from necrotic enterocolitis and he was operated on and 10 cm of the small intestine, at a distance of 15 cm from the ileocaecal valve were removed. Besides that, a colostomy on the right side was performed. Necrotic enterocolitis and respiratory insufficiency syndrome due to the prematurity of the infant constituted the medical co-morbidities of our patient.

Based on this findings, an initial CT scan was performed which verified the aforementioned finding, and an external ventricular drainage was inserted through the frontal horn of the right lateral ventricle, in order to manage the post hemorrhagic ventricular dilation.

A few days after the operation, a CSF leak from the surgical wound was observed and the ventricular catheter was removed, the wound was revaluated and a new EVD was inserted due to fullness of the anterior fontanelle and new onset opisthotonos.

**Fig. A1:**
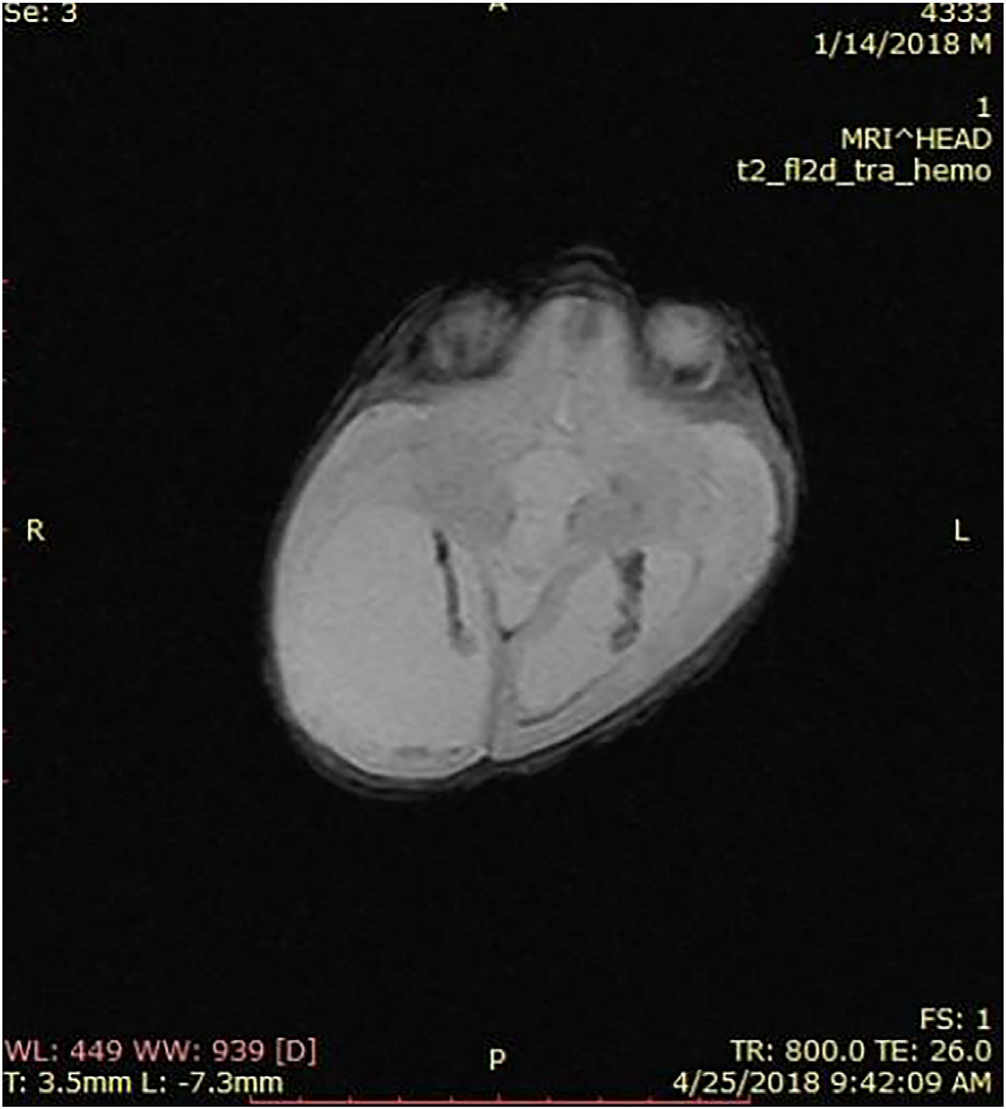
MRI scan, axial view, demonstrating the ventricular enlargement and the hemosiderin deposits within it.

**Fig. A2:**
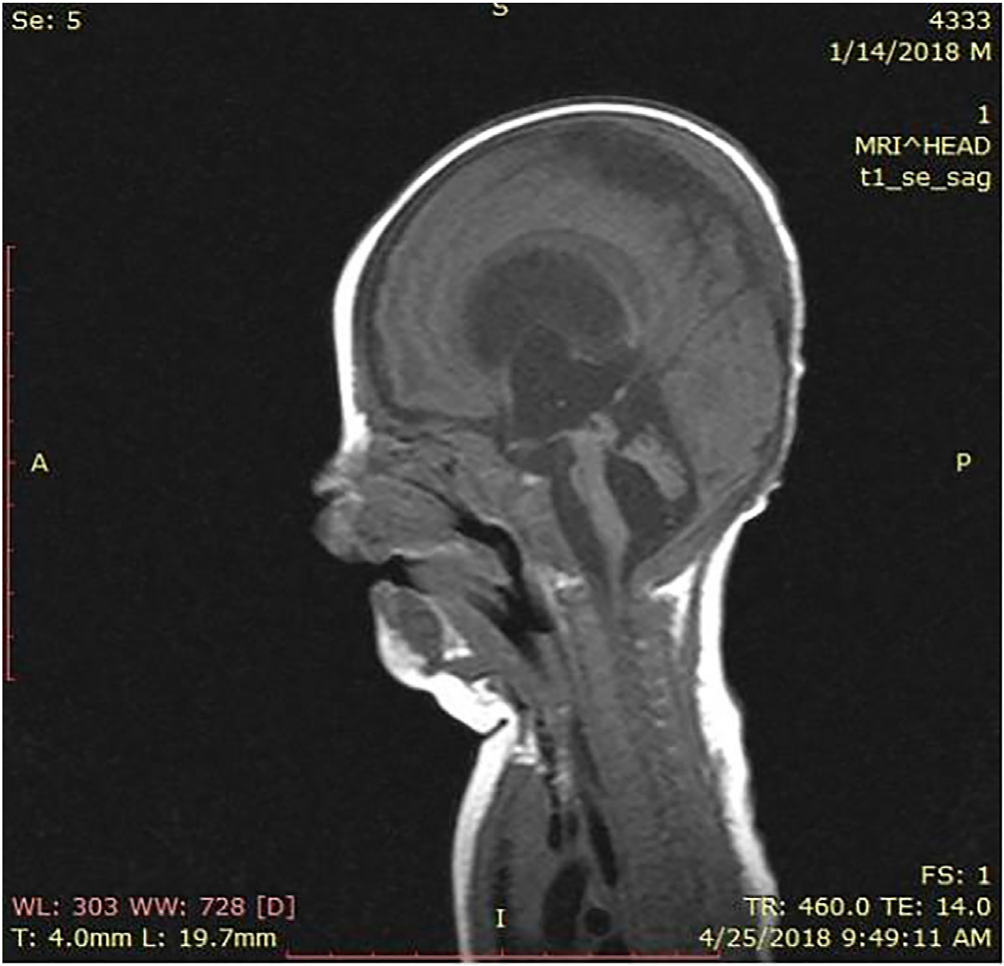
MRI scan, sagittal view, demonstrating the hypoplasia of the cerebellum and of the posterior fossa.

**Fig. A3:**
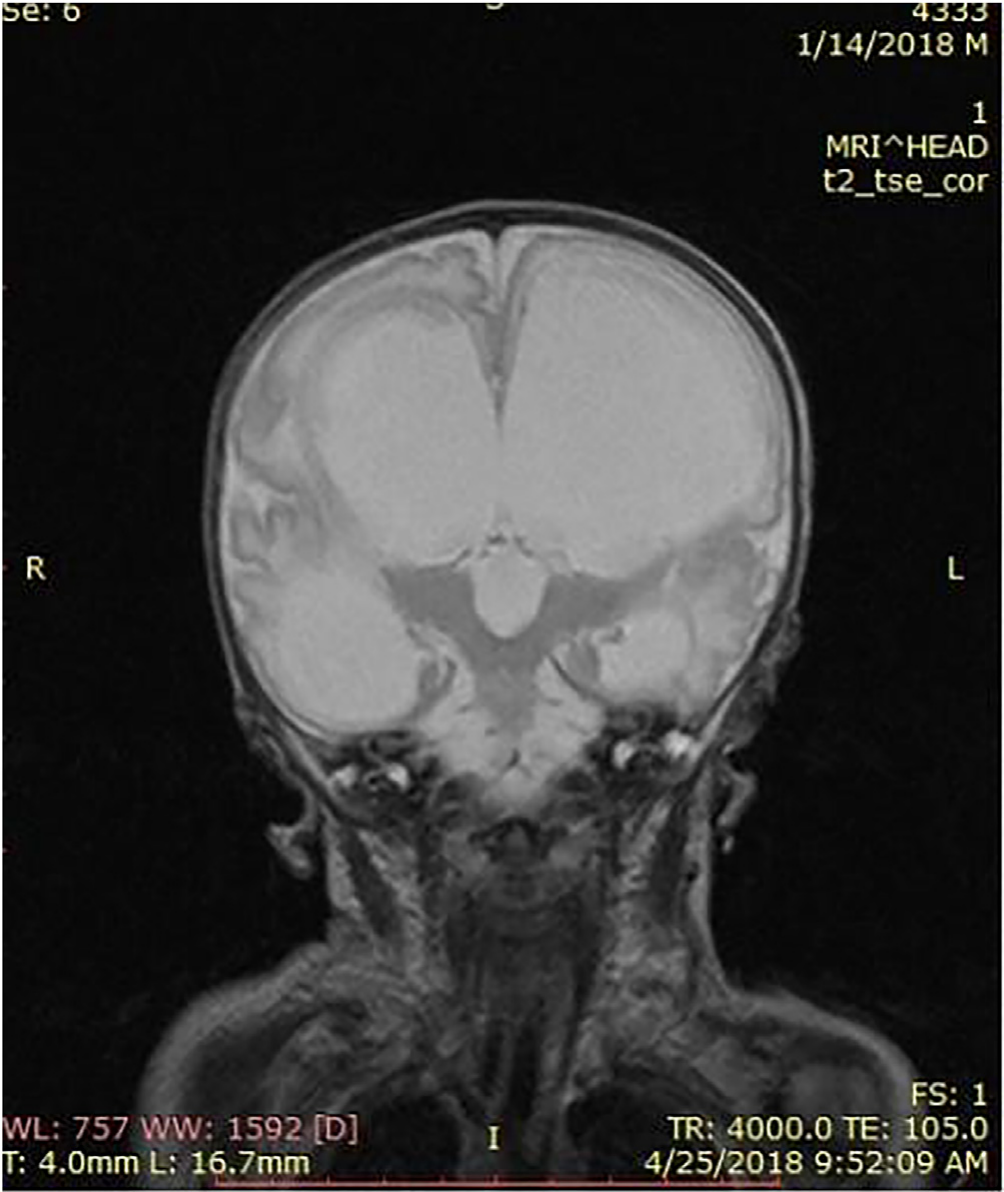
MRI scan, coronal view, delineating the significant enlargement of the frontal horns and the significant gyral hypoplasia.

An MRI was performed to evaluate the course of the post hemorrhagic ventricular dilation∙ it revealed significant dilation of the 3rd, 4th, and lateral ventricles bilaterally, as well as dilation of the anterior subarachnoid space, a finding that could be explained based on the age of the infant. Concurrently, hypoplasia of the pons and cerebellum were identified (Fig. A1, Fig. A2, Fig. A3).

Two weeks after, two months after birth, the EVD was removed and a VP shunt was introduced (non-programmable valve, opening pressure 120 mmHg).

Three weeks after the operation, the abdomen of the infant appeared to distend gradually and signs of increased intracranial pressure appeared, namely reappearance of opisthotonos and fullness of the anterior fontanelle. Abdominal ultrasound revealed significant amount of free floating, non-encapsulated fluid and compatible with dysfunction of the peritoneal segment of the VP shunt. Taking into consideration the medical history of the patient, it was attributed to decreased capacity of the peritoneal cavity to absorb the CSF, in the context of immaturity.

After 10 days, another CSF leak was noticed across the surgical wound and the VP shunt was removed to avoid the possibility of infection. The wound edges were incised; skin and subcutaneous tissues were dissected and sutured again at healthy margins. A few days after, the anterior fontanelle was tense and ultrasound examination revealed that the ventricular size was increased, so a new EVD was inserted.

A new MRI was executed, in order to monitor the radiological appearance of the ventricular system of the infant. We notice that the posterior fossa of the patient is relatively small, with concurrent hypoplasia of the pons and cerebellum. We noticed that the 4th ventricle and its foramens were dilated. The white matter of the cerebral hemispheres was diminished in size with accompanying dilation of the subarachnoid space in the vicinity of the cerebral hemispheres and the posterior fossa.

**Fig. B1:**
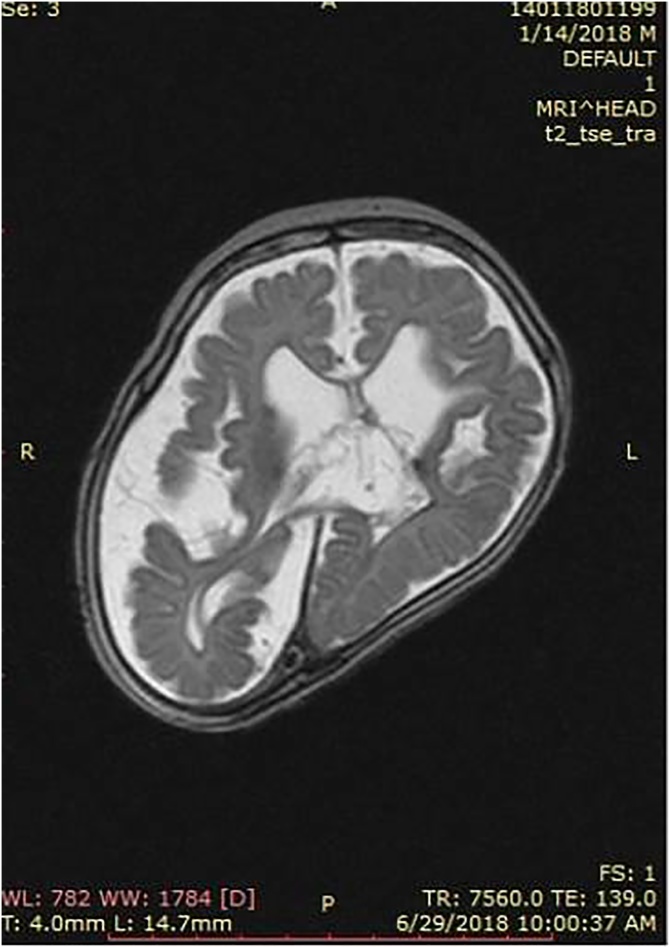
MRI scan, axial view, demonstrating reduction of the size of the ventricles with a degree of dilation of the subarachnoid spaces.

**Fig. B2:**
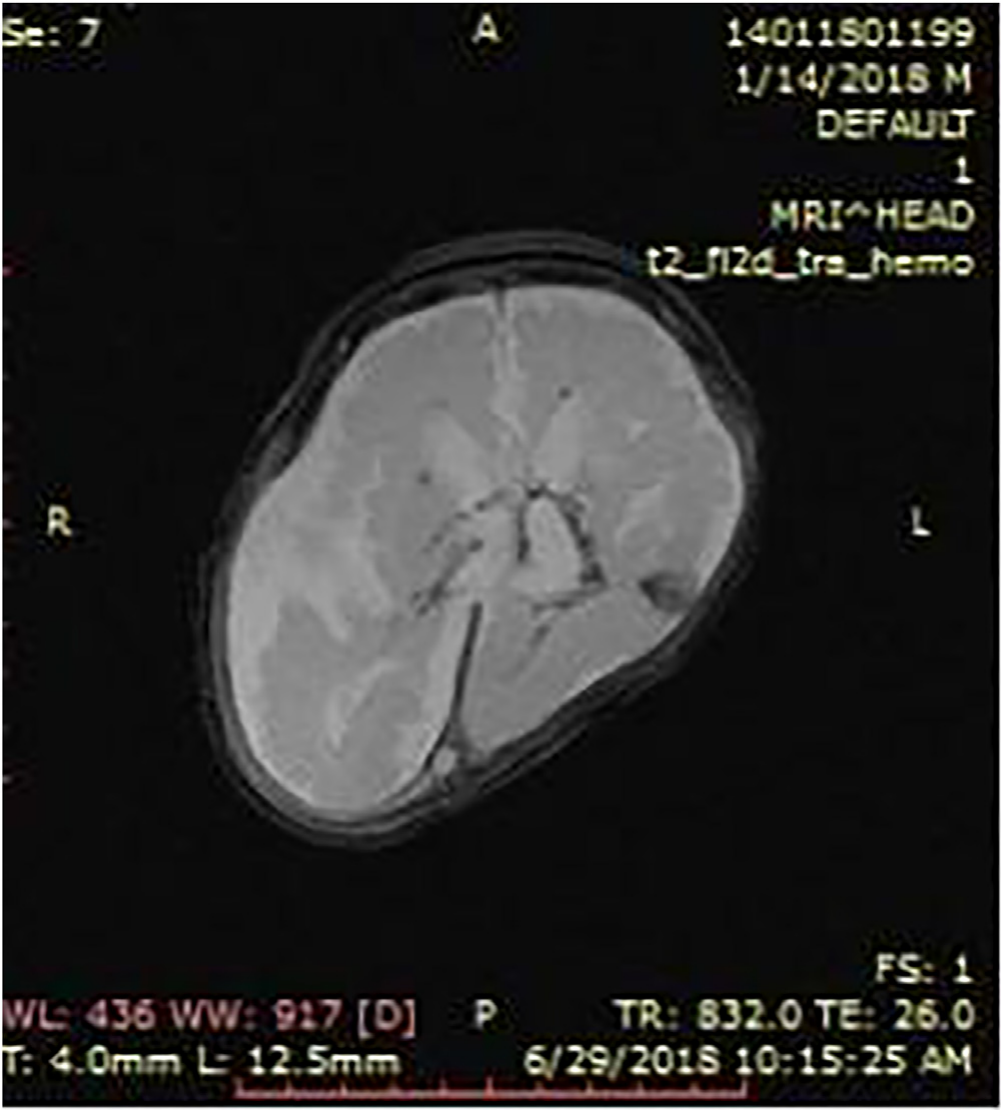
MRI scan, axial view, depicting the remaining hemosiderin deposits within the ventricular system.

**Fig. B3:**
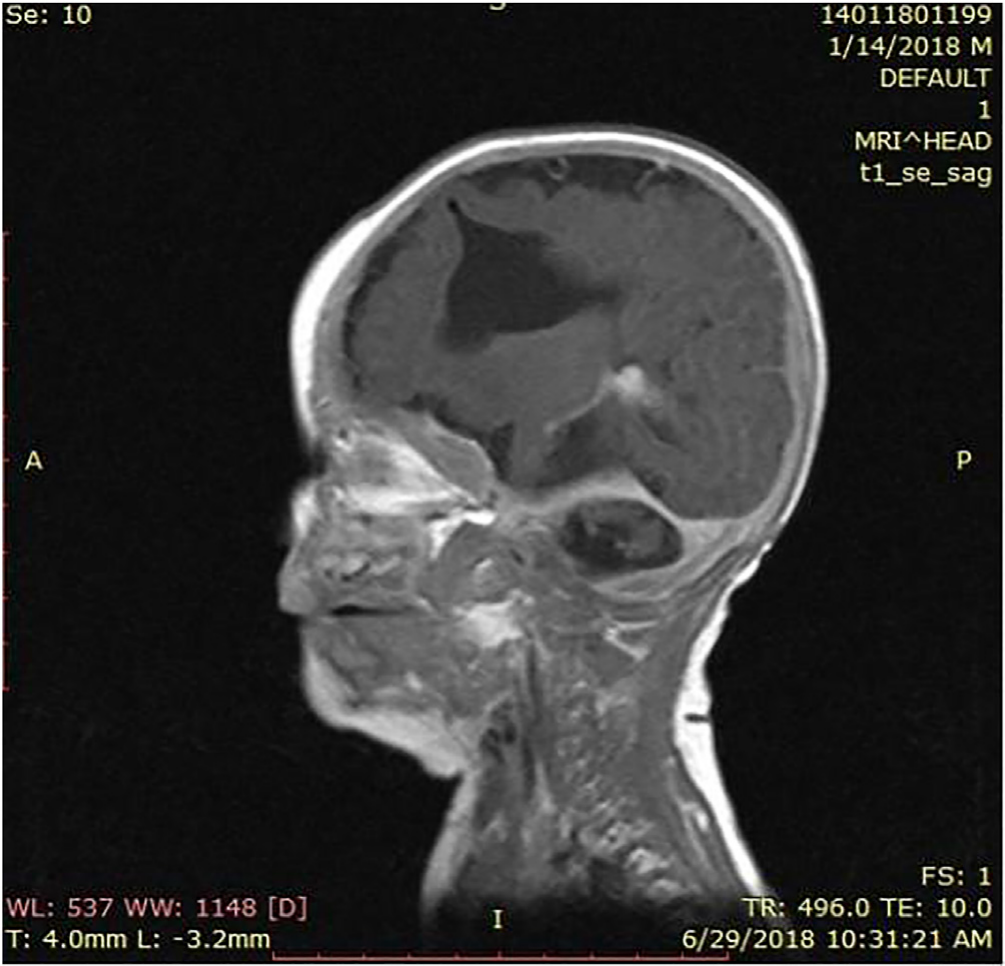
MRI scan, sagittal view, evaluating the hypoplasia of the pons and cerebellum.

We re-evaluated the post hemorrhagic dilation of the lateral ventricles, mainly of their bodies, as well as the hemosiderin deposits in the areas of the choroid plexuses, trigone of the lateral ventricle, caudothalamic sulcus, along the ependymal lining of the lateral and 4^th^ ventricles and also on the surface of the cerebellar hemispheres. These were of low signal intensity on T2 GRE sequence and are attributable to hemorrhage of the germinal matrix layer. Also, we noticed low signal intensity areas at the tentorium cerebelli and falx cerebri on T2 GRE sequence, attributed to subarachnoid hemorrhage (Fig. B1, Fig. B2, Fig. B3).

Three weeks later, a new VP shunt was inserted (opening valve pressure 120 mmHg). After three weeks, we realized that abdominal distension reappeared. Ultrasound of the abdomen verified that there was an excessive CSF collection intraperitoneally, which should be correlated with the inability to absorb the accumulating CSF. The neurological examination of the infant revealed hypotonia of the trunk, hypertonia of the lower extremities and opisthotonos.

We externalized our VP shunt just as a temporary measure, until an ultimate operation was perform to address the abnormalities of CSF circulation. One alternative would be the insertion of a ventriculo-atrial shunt.

Unfortunately, Hickman catheters were inserted to both internal cerebral veins, at different time periods, due to long hospital stay and prematurity of the infant. When an ultrasound of the internal cerebral veins was performed, to evaluate their patency, we identified that no blood flow could be documented to both of them, although a collateral venous network was visible.

Consideration also could be given to performing a ventriculo-pleural shunt. Unfortunately, it is well known that due to connective tissue adhesions in the pleural cavity, repeated operations were performed to keep the tube shunting. Therefore, this procedure was discontinued and we were not convinced that it was the best option for our patient.

Based on that evidence, we decided to perform a Torkildsen operation to our patient∙ size of the subarachnoid space at the cisterna magna and upper cervical level, and presence of any vascular anomalies or abnormal flow void were checked beforehand. We decided to follow the previously described therapeutic plan for our patient as in most of premature infants the absorptive capacity of the peritoneum is gradually regained and we can commonly resolve the problem of hydrocephalus by inserting a ventriculoperitoneal shunt. As soon as it was obvious that the peritoneum was unable to absorb the excess of CSF, we were convinced that Torkildsen shunt should be used as a salvage procedure.

**Fig. C1:**
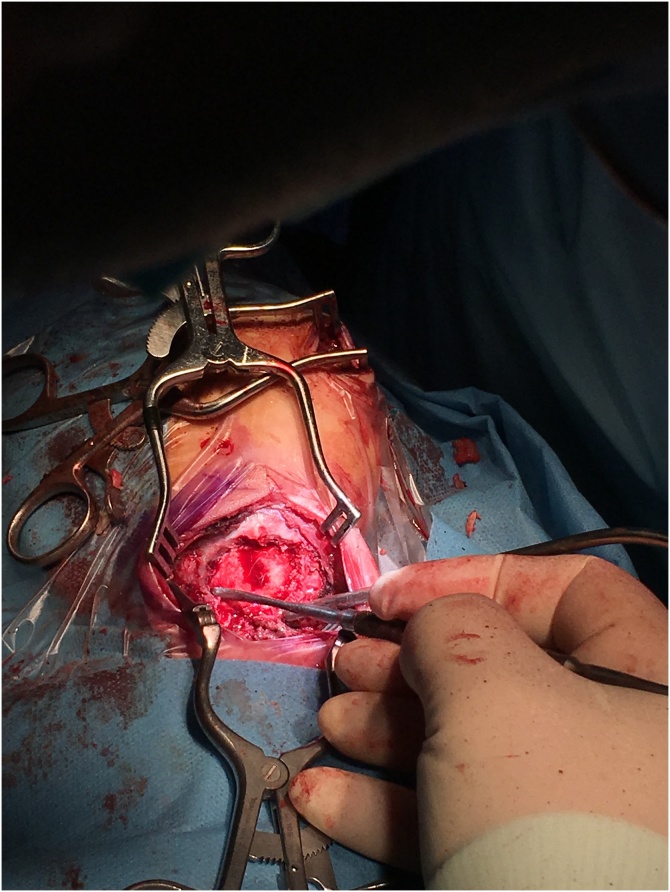
Intraoperative photograph, visualizing the small suboccipital craniectomy performed in order to identify the underlying cisterna magna.

**Fig. C2:**
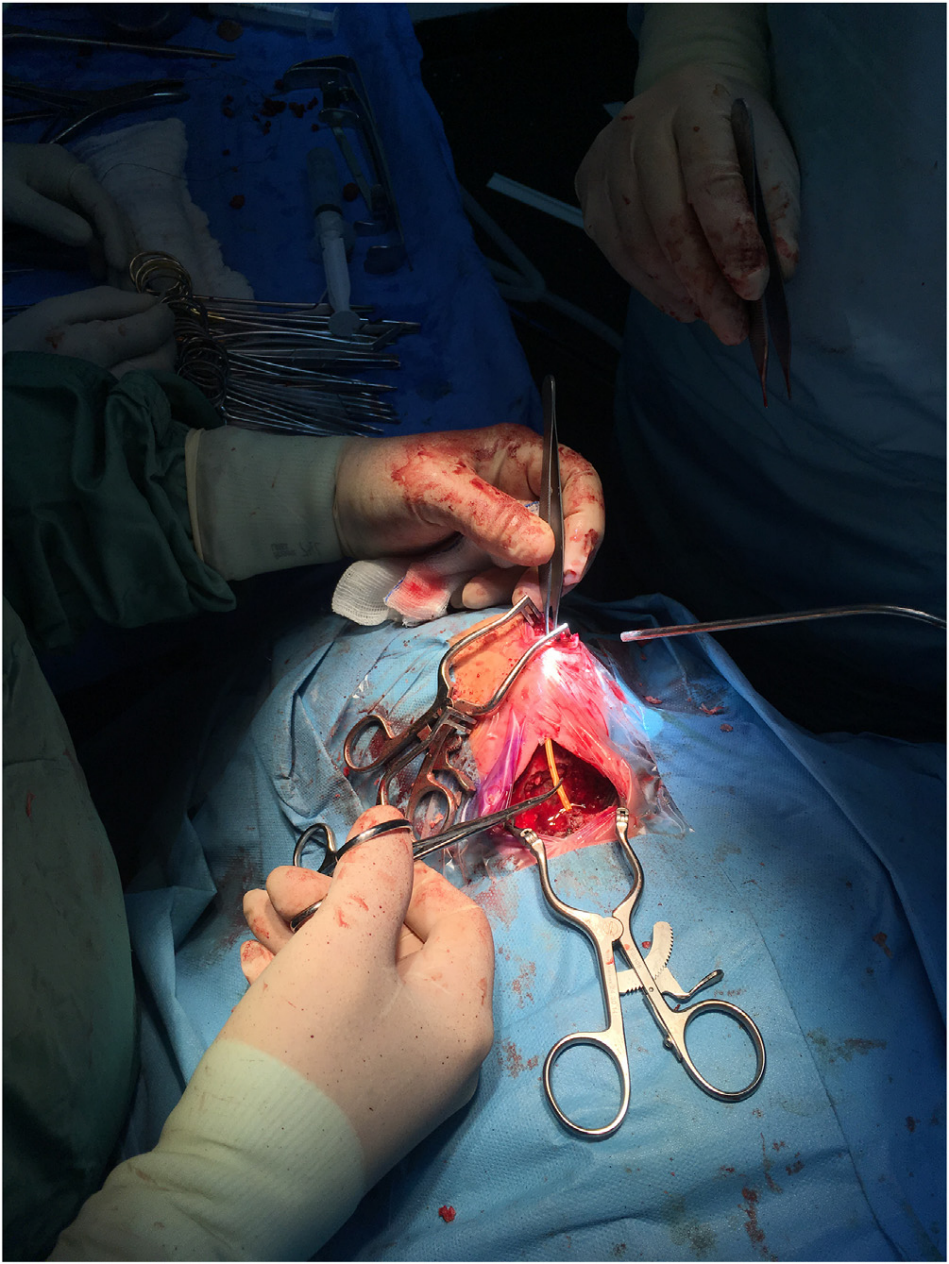
Intraoperative photograph, visualizing the subcutaneous passage of the tube from the occipital horn to the region of the cisterna magna.

A bur hole at the right parieto-occipital region was performed and a ventricular catheter was inserted into the ipsilateral occipital horn. A limited suboccipital craniectomy was performed, the cisterna magna was identified, and the previously inserted ventricular catheter was subcutaneously guided in the vicinity of the cisterna magna. It was subsequently opened and the end of the ventricular catheter was inserted within it and secured for safety (Figs. C1, 2).

**Fig. D1:**
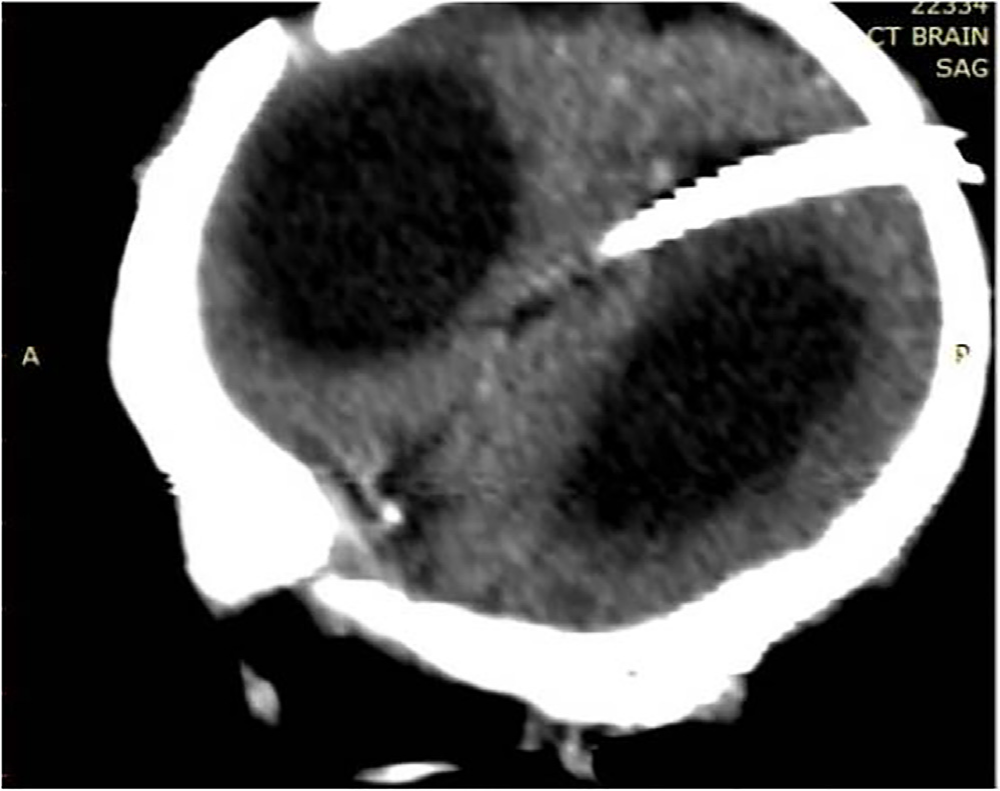
Postoperative CT scan, demonstrating the end of the tube passing through the occipital horn.

**Fig. D2:**
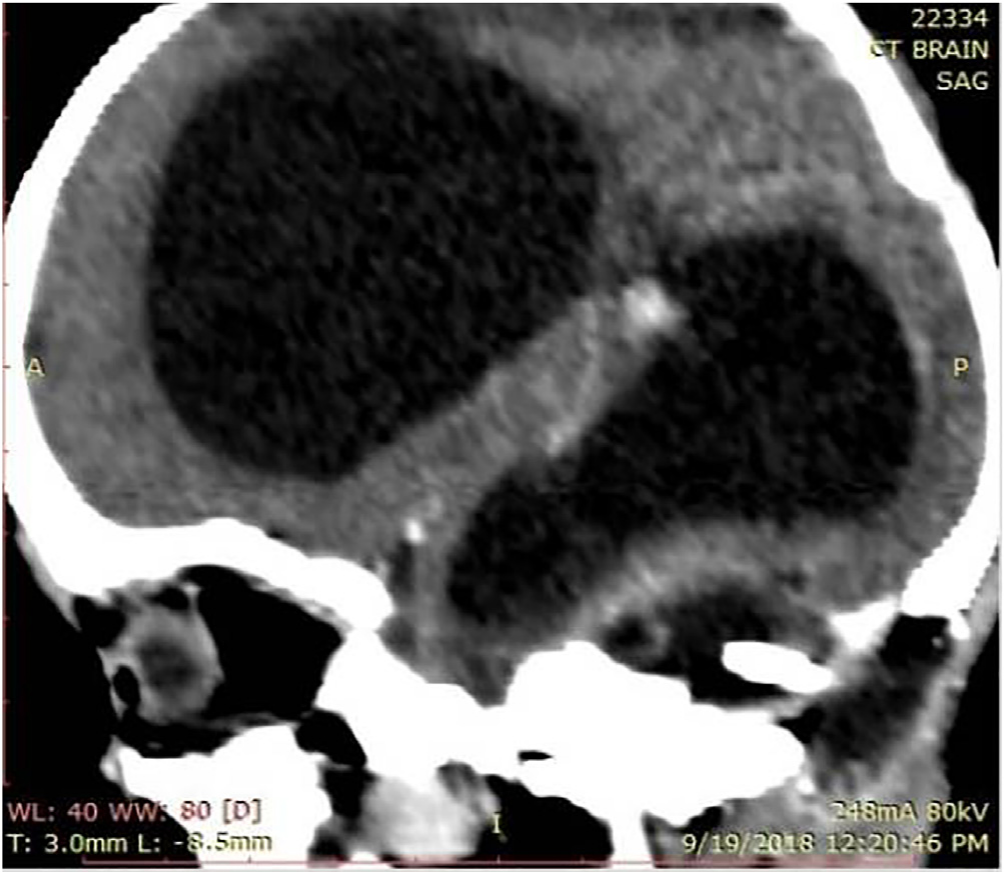
Postoperative CT scan, demonstrating the entry of the other end of the tube into the cisterna magna.

**Fig. D3:**
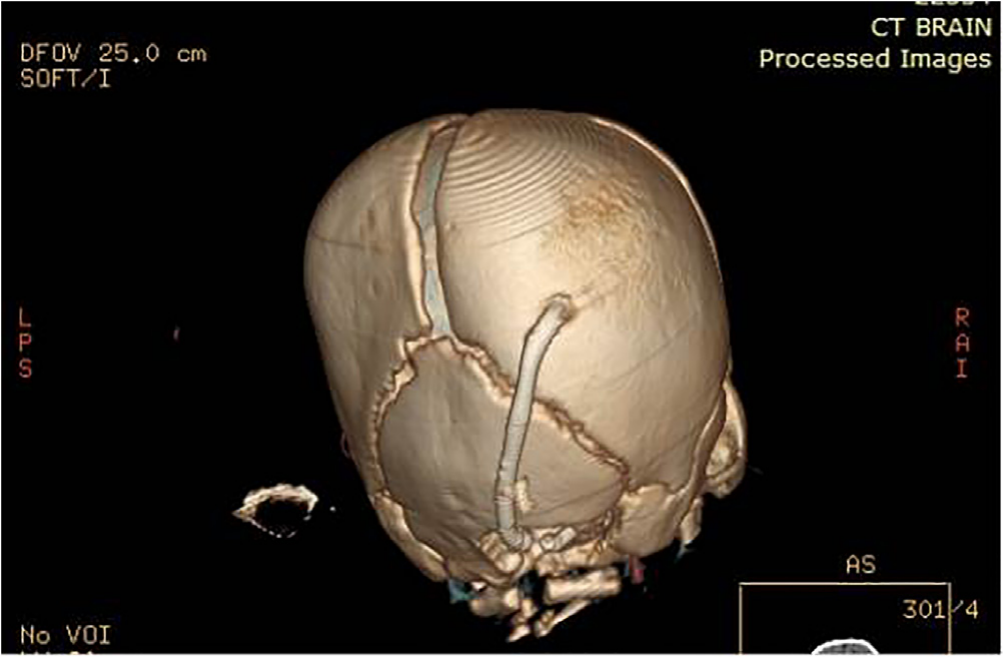
3D CT scan, depicting the course of the inserted tube from the occipital horn to the cisterna magna and its course superficial to the bone.

Immediately after the operation, the episodes of CSF leak were stopped, opisthotonos disappeared and the anterior fontanelle was never under tension. We performed a postoperative CT scan at two weeks’ time, which verified the correct positioning of our catheter and a small reduction in the dimension of the ventricular system (Fig. D1, Fig. D2, Fig. D3). Indeed, we did not expect a dramatic reduction of the ventricular size because due to excessive prematurity the cortex of the infant was excessively underdeveloped and it was largely replaced by CSF. That component of excess CSF could not be handled by any kind of CSF diversion procedure, as it can be seen even when external ventricular drains were used. After that, the ventricular size was evaluated regularly with ultrasound via the anterior fontanelle, which did not demonstrate any significant change in the imaging of the ventricular system. The overall clinical, neurological and ultrasound follow –up extends to a six month period after Torkildsen operation.

## 3 Discussion

Arne Torkildsen was a pioneering Norwegian neurosurgeon best known for developing ventriculocisternostomy (VCS), the first clinically successful procedure for shunting of CSF. It is widely accepted that Torkildsen developed a procedure that received international recognition and was successfully and safely executed by many neurosurgeons at a period of time when no other safe and effective treatment existed for patients with hydrocephalus [5]. Indeed, the Torkildsen shunt appears to have been excessively used as a new surgical procedure for treating hydrocephalus in the 1940s and 1950s [6].

The surgical procedure for the Torkildsen shunt is simple, but could be technically more demanding than VP shunt. The Torkildsen shunt is a simple tubing between two CSF spaces to bridge them. It does not require any special shunt valve to control draining pressure. Patency of the shunt is unable to confirm, demonstrated mild to moderately decrease in ventricular size within a few weeks after the surgery strongly support the Torkildsen shunt played a key role in improvement of clinical findings. One advantage of the Torkildsen shunt is that it enables drainage of CSF through a physiologic pathway, thus avoiding the complications of shunting CSF into the peritoneal cavity.

The Torkildsen shunt can be withdrawn by body growth like a VP shunt. It should be reminded that the distance between head and neck is quite shorter than that between head and abdomen. The distance does increase as a child grows but the influence of body growth to the Torkildsen shunt would be less significant than that happens in children with VP shunt. If the Torkildsen shunt is installed in infants or young children, the shunt withdrawal which requires shunt revision would occur in the future.

## 4 Conclusion

Although this operation is largely abandoned in modern era, we can support the concept that the Torkildsen shunt is a valuable salvage surgical procedure in definite cases of hydrocephalus related to obstruction within the ventricular system [6]. More experience with use of this technique to selected cases and a longer follow-up period, in the order of several years, would be prerequisite for the final re-evaluation of this procedure. According to our opinion, it appears to be worth trying for unusual situations of hydrocephalus, not amenable to other forms of treatment.

## Conflicts of interest

All authors state that we do not have any financial and personal relationships with other people or organisations that could inappropriately influence (bias) our work.

## Sources of funding

There are no sources of funding for our research. Also we declare that no sponsors have any role to our study.

## Ethical approval

Our study is not considered to be Research study, so ethical approval is not applicable.

## Consent

We state that we have parental, written and signed, consent on behalf of the patient to publish this case report.

## Author contribution

Dimitrios Panagopoulos, Corresponding Author, contributed to the paper as follows:

• Concept of design.
• Data collection.
• Analysis and interpretation of data.
• Writing and final approval of the manuscript.

Marios Themistocleous contributed to the paper as follows:

• Concept of design.
• Data collection.
• Writing and final approval of the manuscript.

Andreas Mitsios contributed to the paper as follows:

• Data collection.
• Analysis and interpretation of data.
• Writing and final approval of the manuscript.

Katerina Apostolopoulou contributed to the paper as follows:

• Data collection.
• Analysis and interpretation of data.
• Writing and final approval of the manuscript.

Georgios Sfakianos contributed to the paper as follows:

• Analysis and interpretation of data.
• Writing and final approval of the manuscript.
• Data collection.

## Registration of research studies

Our case report did not did not involve human participants and it does not have to be registered in a publicly accessible database (This section is not applicable to our manuscript).

## Guarantor

I, Dimitrios Panagopoulos, the Corresponding Author of this manuscript, accept full responsibility for the work and the conduct of the study, had access to the data, and controlled the decision to publish.

## Provenance and peer review

Not commissioned, externally peer reviewed.
